# Risk-Based Prenatal Hepatitis C Testing Practices and Results, Alaska 2013-2016

**DOI:** 10.1155/2019/8654741

**Published:** 2019-06-02

**Authors:** Leisha D. Nolen, Courtney Gustin, Sara Seeman, Neil Murphy, Sarah Truitt, Sarah Schillie, Michael G. Bruce, Dana Bruden, James Tiesinga, Brian McMahon

**Affiliations:** ^1^Arctic Investigations Program, Division of Preparedness and Emerging Infections, National Center for Emerging and Zoonotic Infectious Diseases, Centers for Disease Control and Prevention, USA; ^2^Obstetrics and Gynecology, Southcentral Foundation, Anchorage, AK, USA; ^3^Division of Viral Hepatitis, National Center for HIV/AIDS, Viral Hepatitis, STD, and TB Prevention, CDC, USA; ^4^Alaska Native Tribal Health Consortium, Anchorage, AK, USA

## Abstract

Hepatitis C virus (HCV) infection in pregnant women is of concern as it presents a health threat not only to the mother, but also to her infant. A retrospective analysis was performed to evaluate HCV testing and exposure in women who delivered infants between 2013 and 2016 at a referral hospital in Alaska. Multiple risk behaviors were evaluated, including drug dependency or abuse (drug abuse), tobacco use, alcohol dependency or abuse, and late presentation to prenatal care. Of the 2856 women who delivered between 2013 and 2016, 470 (16.5%) were tested for HCV during pregnancy and 1356 (47.5%) were tested at any time prior to delivery (including pregnancy); 62 (2.2%) were positive for HCV antibodies. Of the 162 women with a documented history of drug abuse, 95 (58.6%) were tested for HCV during pregnancy and 143 (88.3%) were tested at any time prior to delivery (including pregnancy); 30 (18.5%) were positive for HCV antibodies. Forty-nine women (34%) with a documented history of drug abuse who were not previously known to be HCV positive were not tested for HCV during their pregnancy. In conclusion, approximately 2% of pregnant women in the study population were known to have been exposed to HCV by the time of their delivery. One-third of women with documented drug abuse did not have an HCV test during pregnancy, revealing gaps in HCV testing of pregnant women. Further studies are needed to understand the full costs and benefits of risk-based screening versus universal screening in this and other populations.

## 1. Introduction

Hepatitis C virus (HCV) can cause chronic infection that can lead to liver failure and cancer. Most people who are infected are not aware of their infection because they do not have any symptoms [1], leaving a large number of people at risk of significant liver disease. HCV is a concern to the obstetrics provider as it can be transmitted from mother to child around the time of birth. It is estimated that between 4-6% of babies born to HCV positive mothers develop HCV infection [2, 3]. Currently there is no effective strategy to prevent HCV transmission from mother to baby [4]. Prenatal clinical care visits represent an opportunity to identify not only women who would benefit from treatment for HCV after pregnancy, but also a way to identify infants who should be evaluated for HCV infection.

National guidelines only recommend HCV testing for high risk populations, including all persons born between 1945 and 1965. Testing is recommended in people outside this age range if they have risk factors including IV drug use (IDU), incarceration, unregulated tattooing, blood transfusion prior to 1992, and unprotected sex with high risk individuals [5, 6].

Recent reports have found an increased prevalence of HCV infection in reproductive aged women in the U.S. [7–11]. In Alaska there has been a 100% increase in the number of reported cases of HCV in persons ages 18-29 between 2011 to 2015 [10]. Women represented 53% of the cases in this age group. Studies have shown that risk-based screening strategies miss a significant number of HCV positive women [12–15]. Recently, two national medical organizations, American Association for the Study of Liver Diseases and the Infectious Diseases Society of America, have released recommendations that all pregnant women should be tested for HCV [16]. Here we report an in-depth investigation into the risk-based HCV testing practices and results in an OB clinic.

## 2. Materials and Methods

An evaluation of HCV-exposure and testing in women who gave birth was initiated by the obstetrics clinic at an Alaska Native Tribal health system referral hospital. Women who had delivered between January 1, 2013, and December 31, 2016, were identified through the hospital's electronic medical record (EMR). In order to ensure that all test results were captured, the analysis only included women who lived in the region where both inpatient and outpatient tests were recorded in the EMR. The EMR was queried for demographic information and all recorded HCV testing dates and results since January 2010. The hospital laboratory algorithm requires that any sample that tests positive for HCV antibodies (anti-HCV) is automatically tested for HCV RNA by PCR. A woman with a positive anti-HCV test was considered HCV-exposed, while a woman with a positive HCV RNA PCR was considered infected. International classification of disease (ICD) diagnosis 9 and 10 codes were used to tobacco use, alcohol dependency or abuse, and late presentation to prenatal care (LPPC) (Drug Depency or abuse: 304.4, 304.41, 304.43, 304.6, 304.61, 305.7, 305.71, 305.72, 305.73, 304.2, 304.21, 304.23, 305.6, 305.62, 305.63, F14.10, F14.21, F14.90, 304.01, 304.02, 304.03, 304.7, 304.71, 304.72, 304.73, 305.5, 305.51, 305.52, 305.53, F11.10, F11.129, F11.20, F11.21, F11.23, F11.288, F11.90; Alcohol Use: 305.0^+^, 303.*∗∗*, F10.*∗∗∗*; Late to prenatal care: V23.7, O09.30; Smoking: 305.1, F17.*∗∗∗*, O99.33*∗* (^+^ can be 0-3, *∗* can be 0-9).). As ICD codes specific for IDU do not exist, the ICD codes related to drug dependence or abuse (drug abuse) of opiates, cocaine, and amphetamine were used as proxies. Women were considered positive for the risk factor if ≥ 1 ICD code was recorded for that risk factor. Tobacco use and alcohol dependency were only evaluated for the two years prior to delivery; IDU was evaluated from the beginning of the EMR (January 2010). LPPC used the standard definition of beginning prenatal care after the end of the second trimester of pregnancy. Prenatal testing was defined as any test that occurred between 270 days prior to and 7 days after the delivery date. Statistical analysis was performed using SAS 9.4 (Cary, NC). Proportions between groups were compared using the chi-square test for univariate analysis. Multivariate analysis was conducted using logistic regression and odds ratios are reported for effect sizes. Statistical significance was defined as p<0.05 and 95% confidence intervals (CI) are reported.

## 3. Results

**Figure 1:**
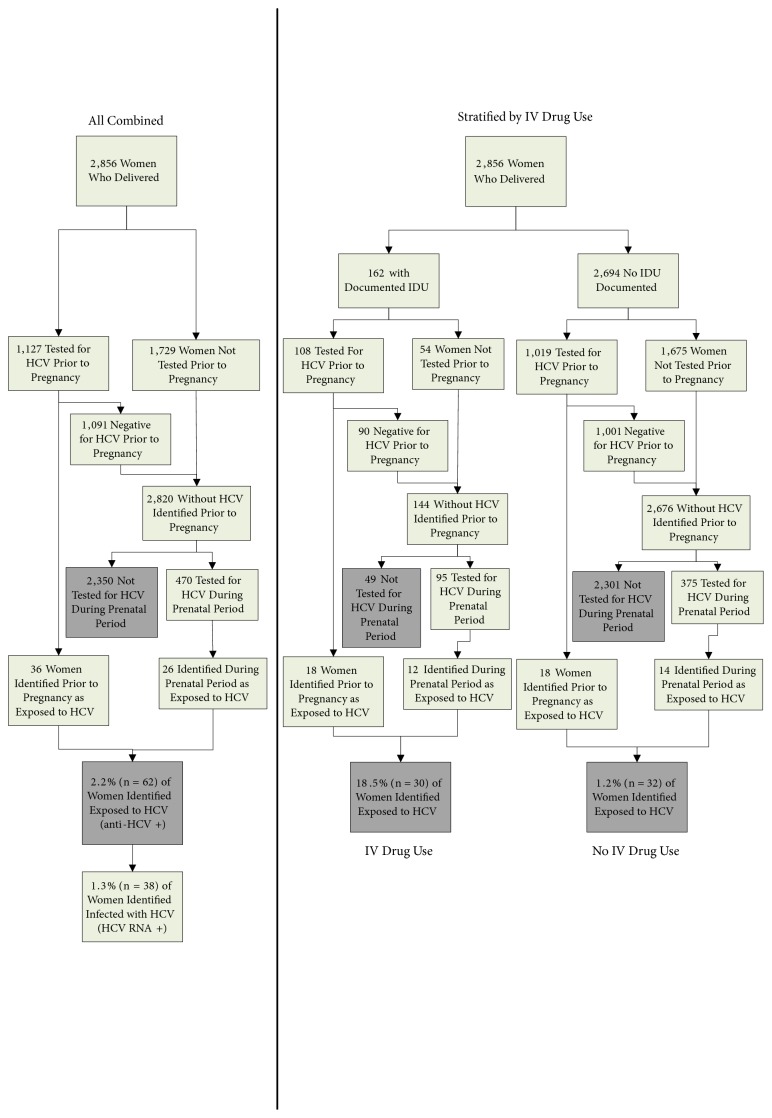
Flow diagram of those included in the evaluation to determine exposure to hepatitis C among women who delivered infants, 2013-2016. Gray boxes highlight the outcomes of interest.

A total of 2856 women from the study area gave birth in the hospital during the study period (Figure 1). Of these, 470 (16.5%) were tested for HCV during their prenatal period and a total of 1356 (47.5%) were tested at any time prior to delivery (either during or prior to pregnancy). Thirty-six women had tested positive for exposure to HCV prior to their prenatal period and 26 new HCV-exposed women were identified based on prenatal testing, resulting in 62 (2.2%, 95% CI: 1.6, 2.6) women who had been exposed to HCV by the time of their delivery. Thirty-eight of the 62 women (1.3%, 95% CI: 0.9, 1.7) were infected (HCV RNA positive).

In multivariable analysis, factors associated with increased odds of HCV exposure were documented drug abuse (OR 12.4, CI 7.0-21.9, p<0.0001), tobacco use (OR 2.0, CI 1.1-3.6, p=0.02), LPPC (OR 3.5, CI 1.9-6.3, p<0.0001) and increased age at the time of delivery (women 35 years of age or older had higher odds compared to <25 years of age (OR 3.1, CI 1.3-7.6, p=0.01) Table 1).

A total of 162 (5.7%) pregnant women in the study had one or more ICD codes for drug abuse (Figure 1). Eighteen of these were previously known to be HCV exposed. Ninety-five of the remaining women (66%) were tested prenatally for HCV and 143 were tested at any time prior to delivery (88.3%). Twelve women with a history of drug abuse were newly identified by prenatal testing as having been HCV exposed, resulting in a total of 30 (18.5%) HCV-exposed women with a history of drug abuse by the time of delivery. Forty-nine (34%) women with documented drug abuse and unknown HCV status were not prenatally tested for HCV. When women with a history of drug abuse who were tested prenatally were compared to those who were untested, significantly more had also been tested prior to pregnancy (100% vs 64%, p,0.0001) and no difference was found between the time since their most recent pre-pregnancy HCV test (mean 783.7 vs 739.6 days prior to delivery, respectively), nor the type of drug abuse code recorded (p=0.14). There was a statistically significant difference between the duration of time since the last drug abuse ICD code (median 14 vs 66 days prior to delivery, respectively, p=0.005); however 31 (63.3%) women who were untested prenatally had an ICD code for drug abuse during their pregnancy window. There were more occurrences of ICD codes related to drug abuse in the tested versus the untested drug abuse women (mean 5.1 vs 3.8, respectively, p=0.0037).

Of the 2694 women without documented drug abuse, 18 were known to be HCV-exposed prior to pregnancy. Of the 2676 remaining women, 375 (14.0%) were prenatally tested and fourteen new HCV-exposed women were identified. Overall, 32 (1.2%) women with no documented history of drug abuse were HCV-exposed by the time of their delivery. For women with no history of drug abuse, HCV exposure was associated in multivariable analysis with LPPC (OR 4.5, CI 2.0-10.0, p=0.0002), tobacco use (OR 2.6, CI 1.2-5.7, p=0.01), and increased age (p=0.03); women with 35 years of age or older had higher odds compared to <25 years of age (OR 5.4, CI 1.6-18.6, p=0.008) (Table 1).

## 4. Discussion

This study found a high prevalence of HCV exposure in new mothers and also identified missed opportunities to test high-risk women. Overall, 2.2% of women included in this study were positive for anti-HCV, compared to a national estimate of 1.4% of the general population [17]. The percentage identified here is likely an under-estimate, as only 47% were ever tested for HCV. Women with a history of drug abuse had the highest odds of being exposed to HCV, yet 34% of women with documented drug abuse history were not prenatally tested for HCV.

National recommendations support testing for HCV infection among all individuals with a history of IDU [6]. This study found that a third of the women with ICD codes suggestive of documented IDU (drug abuse) were not prenatally tested. Within the drug abuse population, the prenatally tested and untested women did not differ with regard to the time of their pre-pregnancy HCV test or the type of drug recorded. Two characteristics were statistically different between the tested and untested population: the number of drug abuse ICD codes and the duration of time since the last drug abuse ICD code. While these values are statistically different for the two groups, medically the difference in the numbers does not reflect a difference in risk. Both these groups of women should be considered at risk as any IDU is considered a risk factor for HCV exposure. Importantly, 63% of women who were not tested had a code for drug abuse recorded in their chart during their pregnancy, indicating a clinician documented drug abuse in the medical records but did not order an HCV test. Based on the similarity between the two groups, we hypothesize the percent positive of untested women will be similar to that in the tested women (19%). If correct, an estimated nine HCV exposed women with documented drug abuse were missed secondary to a lack of screening.

IDU is considered the most important risk factor for HCV exposure [18]. Unfortunately, no ICD codes exist for IDU; thus this and other studies are limited in their ability to evaluate this important risk factor. Using ICD codes for drug abuse and dependency as proxies for IDU will give an overestimation of IDU as people can have addition or dependence to these substances without injecting drugs. Even with this limitation in mind, this study found 32 (1.2%) women who had no documented drug abuse history had a positive test for HCV. These women may have been exposed to HCV through other risk behaviors, such as tattooing at an unregulated location, incarceration, and intranasal drug use [18]. A second possibility is that these women did not reveal their history of drug abuse to the clinician or it was not recorded. drug abuse may be underreported during pregnancy due to stigmatization or maternal fear of losing custody of the child. Women who were HCV-exposed and did not have an ICD code for drug abuse were more likely to smoke, be older, and present late to prenatal care. While the first two of these factors are very common and thus difficult to use as risk markers, late presentation to prenatal care could possibly be used as a marker for increased risk of HCV exposure for clinicians.

This study has several limitations. The study hospital specifically serves the American Indian/Alaska Native population, which may have different risk factors or exposures than other U.S. populations; therefore these results may not be true for the general population of U.S. pregnant women. Another limitation is that it was not possible to evaluate information that was not recorded in the medical record. For example, incarceration and unregulated tattooing are known risk factors for HCV infection; however it was not possible to collect this information from the EMR. As a result, we were unable to evaluate testing in women who have these risk factors. One additional limitation is our inability to evaluate risk factors that the physician did not document with an ICD code. This limitation would result in underestimation of the number of women stratified as at risk in our analysis.

This investigation highlights a population of pregnant women in Alaska who are at risk for HCV infection and also reveals gaps in risk-based HCV testing of pregnant women. All of the women included in this study are within a health care system that can test for HCV without cost to the patient; thus no cost or laboratory barriers can explain the lack of testing that was observed. It is important that all in the medical community recognize the importance of screening at-risk individuals when they present for care. Further studies are needed to understand the barriers to appropriate testing and the full costs and benefits of risk-based screening versus universal screening in this and other populations.

## Figures and Tables

**Table 1:** Raw and adjusted odds ratios for documented exposure to hepatitis C among women who delivered infants, 2013-2016.

Risk Factor	Level	% Known HCV Exposure	Univariate Results		Multivariate Results	
			Odds Ratio	P-value	Odds Ratio	P-value
		(n/N)	[95% CI]		[95% CI]	
All Mothers						

Age at delivery (in years)	< 25	1.2% (11/945)	ref	0.01	ref	0.049
	25 – 29	2.5% (23/926)	2.2 [1.0, 4.5]		2.0 [0.9, 4.3]	
	30 - 34	2.7% (17/632)	2.3 [1.1, 5.0]		2.7 [1.2, 6.1]	
	≥ 35	3.1% (11/353)	2.7 [1.2, 6.1]		3.1 [1.3, 7.6]	

Injection Drug Use	Yes	18.5% (30/162)	18.9 [11.2, 32.1]	<.0001	12.4 [7.0, 21.9]	<.0001
	No	1.2% (32/2694)	ref		ref	

Tobacco Use	Yes	5.3% (23/433)	3.4 [2.0, 5.8]	<.0001	2.0 [1.1, 3.6]	0.02
	No	1.6% (39/2423)	ref		ref	

Alcohol Use	Yes	3.9% (6/156)	1.9 [0.8, 4.5]	0.15	Removed from model	
	No	2.1% (56/2700)	ref			

Late to Prenatal Care	Yes	7.9% (22/277)	5.5 [3.2, 9.4]	<.0001	3.5 [1.9, 6.3]	<.0001
	No	1.6% (40/2579)	ref		ref	

Number of Deliveries	1	1.6% (10/608)	ref	0.01	Removed from model	
	2-4	1.7% (28/1614)	1.1 [0.5, 2.2]			
	≥5	3.8% (24/610)	2.4 [1.1, 5.0]			

Nondrug abuse Mothers						

Age at delivery (in years)	< 25	0.4% (4/902)	ref	0.004	ref	0.03
	25 – 29	1.2% (10/858)	2.6 [0.8, 8.5]		2.9 [0.9, 9.2]	
	30 - 34	1.8% (11/601)	4.2 [1.3, 13.2]		4.8 [1.5, 15.4]	
	≥ 35	2.1% (7/333)	4.8 [1.4, 16.6]		5.4 [1.6, 18.6]	

Tobacco Use	Yes	2.7% (10/371)	2.9 [1.4, 6.2]	0.008	2.6 [1.2, 5.7]	0.01
	No	1.0% (22/2323)	ref		ref	

Alcohol Use	Yes	4.1% (5/122)	4.0 [1.5, 10.6]	0.01	Removed from model	
	No	1.1% (27/2572)	ref			

Late to Prenatal Care	Yes	3.9% (9/231)	4.3 [2.0, 9.4]	0.001	4.5 [2.0, 10.0]	0.0002
	No	0.9% (23/2463)	ref		ref	

Number of Deliveries	1	0.9% (5/582)	ref	0.03	Removed from model	
	2-4	0.9% (14/1538)	1.1 [0.4, 3.0]			
	≥5	2.3% (13/574)	2.7 [0.9, 7.6]			

## Data Availability

This data is not publically available due to the sensitive nature of the information. Our IRB did not feel it was appropriate for this information to be made public.
